# Sustained employment, work disability and work functioning in CKD patients: a cross-sectional survey study

**DOI:** 10.1007/s40620-022-01476-w

**Published:** 2022-10-31

**Authors:** Manna A. Alma, Sijrike F. van der Mei, Sandra Brouwer, Luuk B. Hilbrands, Paul J. M. van der Boog, Herma Uiterwijk, Femke Waanders, Maaike Hengst, Ron T. Gansevoort, Annemieke Visser

**Affiliations:** 1 Department of Health Sciences, Applied Health Research, University Medical Center Groningen, University of Groningen, Groningen, The Netherlands; 2 Department of Health Sciences, Community and Occupational Medicine, University Medical Center Groningen, University of Groningen, Groningen, The Netherlands; 3 Department of Nephrology, Radboud University Medical Center, Nijmegen, The Netherlands; 4 Department of Nephrology, Leiden University Medical Center, Leiden, The Netherlands; 5 Dialysis Center Groningen, Groningen, The Netherlands; 6 Department of Internal Medicine, Isala Hospital, Zwolle, The Netherlands; 7 Department of Internal Medicine, Catharina Hospital, Eindhoven, The Netherlands; 8 Department of Nephrology, University Medical Center Groningen, University of Groningen, Groningen, The Netherlands

**Keywords:** Sustained employment, Work functioning, Chronic Kidney Disease, Survey study

## Abstract

**Introduction:**

Kidney failure negatively affects opportunities for work participation. Little is known about work functioning of employed CKD patients. This study investigates work-related outcomes, and examines associations between patient characteristics and employment status.

**Methods:**

We performed a cross-sectional survey study in nine nephrology outpatient clinics in the Netherlands among working age (18–67 years) CKD Stage G3b-G5, dialysis and transplant patients (*n* = 634; mean age 53.4 years (SD 10); 53% male; 47% Stage G3b-G5, 9% dialysis, 44% transplantation). We assessed employment status, work disability, work-related characteristics (i.e., work situation, working hours, job demands), work functioning (i.e., perceived ability to work, productivity loss, limitations in work), work environment (i.e., work accommodations, psychosocial work environment), as well as health status and fatigue.

**Results:**

Sixty-five percent were employed reporting moderate work ability. Of those, 21% received supplementary work disability benefits, 37% were severely fatigued, 7% expected to drop out of the workforce, and 49% experienced CKD-related work limitations. Work accommodations included reduced working hours, working at a slower pace, adjustment of work tasks or work schedule, and working from home. Multivariable analysis of sustained employment showed associations with younger age, male gender, higher level of education, better general and physical health and pre-emptive transplantation. Transplant patients had the highest work ability and highest expectation to maintain work. Dialysis patients had the highest productivity loss and perceived the most limitations regarding functioning in work. Stage G3b-G5 patients reported the lowest social support from colleagues and highest conflict in work and private life.

**Conclusions:**

Employed CKD patients experience difficulties regarding functioning in work requiring adjustment of work or partial work disability. In addition to dialysis patients, stage G3b-G5 patients are vulnerable concerning sustained employment and work functioning.

**Supplementary Information:**

The online version contains supplementary material available at 10.1007/s40620-022-01476-w.

## Introduction

Loss of kidney function negatively affects opportunities for work participation. Kidney replacement therapy, such as dialysis, is often associated with loss of work [1, 2]. Transplantation offers recipients the potential to return to a productive working life, but a substantial proportion of patients remain unemployed [3]. Besides medical treatment, kidney failure in itself is disabling and causes changes in employment, as even late-stage CKD patients may stop working [4].

Previous studies on work participation in CKD patients focused mainly on employment rates. Little is known about how patients perceive their work functioning, their work ability, work productivity and work relationships [5]. Regarding work limitations, a Dutch survey study [6] showed that 85% of employed CKD patients experienced work limitations, in the literature also known as presenteeism, or attending work while ill [7]. Moreover, 40% needed work accommodations, usually a reduction in working hours [5]. Work accommodations and job control may thus facilitate sustained employment [8].

From the patients’ perspective, participation in paid work is highly valued, as it enhances quality of life, provides a sense of identity, and provides financial security [9]. Participation in work was among the ten highest-ranked outcomes prioritized by CKD patients [10]. Nephrology care may have the potential to help CKD patients remain employed, however, currently, work-related issues may not be adequately addressed in the nephrology care [11]. Greater insight into the working life of CKD patients and potential risk groups may help identify effective interventions, may inform targeting of these interventions and as such may enhance the development of a supportive infrastructure enabling patients to remain employed in order to improve their quality of life [11, 12].

To address this knowledge gap, we designed a study that investigates sustained employment, work disability, work functioning, and work environment in CKD patients. In addition, we assess differences between employed and non-employed patients and examine associated patient characteristics of sustained employment, as well as differences between stage G3b-G5, dialysis, and transplant patients concerning work-related outcomes.

## Methods

### Study design and population

**Fig. 1:**
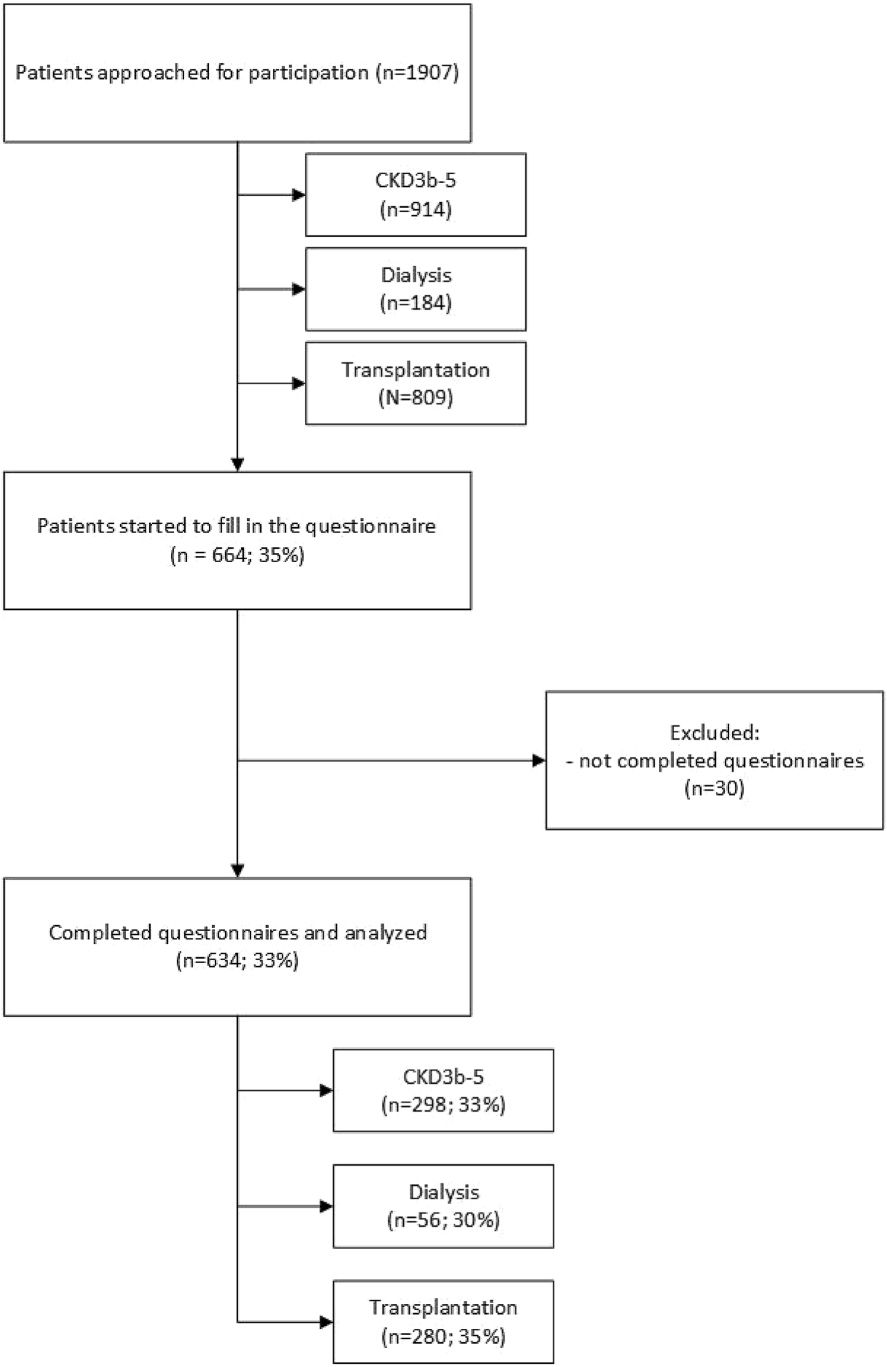
Flow diagram of the inclusion of participants

For this cross-sectional survey study (May–September 2019), nine nephrology, dialysis and transplantation departments in the Netherlands invited CKD Stage G3b-G5 patients of working age (18–67 years) to participate. Patients on kidney replacement therapy (dialysis or transplantation) were included if they had started dialysis or received their transplantation 6 months to 5 years prior to the start of the study. Patients were excluded if they were unable to understand Dutch. To increase the homogeneity of the study population, patients with advanced cancer or heart failure were excluded, as were patients with a life expectancy of less than 1 year as determined by the nephrologist or specialized nurse. Eligible patients were invited by a letter from their nephrologist and consented to participate by completing an online, or if they preferred, a paper questionnaire. Non-responders received a reminder within 3 weeks. Figure 1 presents a flow diagram of the inclusion. For every 50 respondents, a gift voucher of 100 euro was raffled among the respondents.

The study adhered to the Declaration of Helsinki and was approved by the Medical Ethics Review Board of the University Medical Center Groningen (M15.169470).

### Socio-demographic, clinical and health status characteristics

Socio-demographic characteristics assessed were: age, gender, highest attained level of education, ethnicity and financial situation. Clinical characteristics included primary kidney disease, type of dialysis or transplantation, and retransplantation. Comorbidity was assessed with a list of conditions selected by nephrologists (i.e., heart, cerebrovascular, lung or rheumatic disease, diabetes, amputation, epilepsy, migraine, osteoarthritis, permanent injury impairment, depression, other), and the number was categorized as: no comorbidities, 1, and ≥ 2.

We measured general health (1 item), physical functioning (2 items) and mental functioning (3 items) using subscales from the Kidney Disease Quality of Life-Short Form (KDQoL) [13]. Raw scale scores were transformed (0–100 scale). Higher scores indicate better health, physical and mental functioning.

Fatigue was assessed using the fatigue severity subscale (8 items) of the Checklist Individual Strength (CIS) [14]. Items were scored on a 7-point Likert scale (1–7). Higher scores indicate more severe fatigue; a cutoff score > 35 indicates severe fatigue.

### Employment and work disability status, and work-related characteristics

Employment was defined as working for at least one hour per week in a paid position [15]. Full-time students, unpaid caregivers, and those who retired early were considered as non-employed. Work disability was defined as receiving either full or partial work disability benefits administered under Dutch Work Incapacity Acts. After a sickness absence of two years, employees can apply for a disability benefit. Accredited insurance physicians assess the legitimacy of work disability, which can be either full or partial disability, and granted temporarily or permanently [16].

Work-related characteristics included work situation (working according to contract; supplementary work disability benefits; sick leave), and working hours (minor job, < 12 h/week; part-time job, 12–29 h/week; full-time job, ≥ 30 h/week). Job demands were assessed using a self-constructed question (‘What kind of tasks do you perform in your current job?’) with three response options (‘mainly physically demanding tasks’, ‘mainly mentally demanding tasks’, ‘both physically and mentally demanding tasks’).

### Work functioning

We assessed different dimensions of work functioning: work ability, productivity loss, limitations in work performance, and expectation to work in the future [17].

The first item of the Work Ability Index (WAI) asks patients to estimate their current work ability compared with their lifetime best (0 = ‘unable to work’ to 10 = ‘lifetime best’) [18]. This score is classified into: ‘poor’ (0–5), ‘moderate’ (6–7), ‘good’ (8–9) and ‘excellent’ (10) [19].

Productivity loss at work was measured using the quantity item of the Quantity and Quality (QQ) questionnaire [20]. Respondents indicated how much work they actually performed during regular hours on their last regular working day, compared to an average working day before they experienced CKD-related limitations. Response options scaled from 1 (‘practically nothing’) to 10 (‘normal quantity’). Productivity loss based on a regular 8-h working day, was calculated by the formula [(10 – quantity score)/10] × 8 h [20].

Limitations in work performance due to CKD were assessed using a self-constructed question (‘Does the CKD restrict you from performing your job?’) with six response options (1 = ‘no limitations’, 6 = ‘not able to work at all’), and by assessing the type of limitation (e.g., working slower or working fewer hours). In addition, we assessed the expectation to work at 6 months (yes/maybe/no).

### Work environment

We measured various aspects of the work environment: adjustment of work (yes/no), type of provided work accommodations, interpersonal relations, and reactions to the work situation.

Adjustment of work (yes/no) was assessed with a self-constructed question: ‘Have any adjustments been made to your work on your initiative or your employer’s initiative?’ If ‘yes’, respondents could indicate which type of work accommodations were made.

Interpersonal relations (social support by colleagues and by management; recognition) and reactions to the work situation (meaning at work; overall assessment of the psychosocial work environment; job satisfaction; conflict between work-life and private life) were assessed with subscales of the Danish Psychosocial Work Environment Questionnaire (DPQ) [21]. Most items had a 5-points response option (1 = ‘to a very small extent’, 5 = ‘to a very large extent’), whereas the subscales job satisfaction and psychosocial work environment were scored on a scale from 0 (‘lowest possible level’) to 10 (‘highest possible level’). We calculated subscale scores by averaging the transformed (0–100 scale) item scores. Higher scores indicate higher levels of the measured dimension.

### Statistical analysis

Descriptive characteristics were stratified for CKD stage (i.e., Stage G3b-G5, dialysis, transplantation) and for employment status. We performed descriptive analyses, analyzed differences between employed and non-employed patients, and differences in work-related variables between CKD stages, using chi-squared tests and Fisher’s Exact tests (nominal or ordinal variables), and using *t-*tests, one-way ANOVAs, or nonparametric Kruskall-Wallis tests (continuous variables). Because we performed multiple comparisons, the level of statistical significance was set at *P* < 0.01.

Associations between socio-demographic, clinical and health characteristics, and the outcome ‘sustained employment’ (employed vs. non-employed), were examined by logistic regression analysis. After examination of the univariable associations, characteristics with an association of *P* < 0.10 were entered into the multivariable model. Sequentially deleting the characteristic with the weakest association resulted in a final model containing only variables related to the outcome (*P* < 0.05). Presence of multicollinearity was tested by calculating variance-inflated factors. In case of missing data, we followed the questionnaire instructions to calculate (sub)scale scores [13, 14, 18–21]. For self-constructed items, missing data were not imputed. We analyzed data using SPSS, version 26 (SPPS, Inc,. Chicago).

## Results

### Study population

We invited a total of 1907 patients to participate. Questionnaires were completed by 634 patients, consisting of 298 Stage G3b-G5 patients, 56 dialysis patients, and 280 patients with functioning kidney transplants. Of the dialysis patients, 43 (77%) were receiving hemodialysis and 13 (23%) peritoneal dialysis (Table 1). For the majority of transplant patients, it was their primary kidney transplantation (*n* = 249, 90%). Almost half of the transplant patients had received a pre-emptive transplantation (*n* = 128; 46%). Twenty-two percent reported polycystic kidney disease as their primary kidney disease and 64% had one or more comorbidities. Participants’ mean age was 53 years (range 19–67); 53% were men, 34% had only primary education.

**Table 1:** Patient characteristics

Characteristics	All patients (*N* = 634; 100%)	Stage G3b-G5 (*N* = 298; 47%)	Dialysis (*N* = 56; 9%)	Transplantation (*N* = 280; 44%)
KRT	334 (53)			
Hemodialysis			43 (77)	
Peritoneal dialysis			13 (23)	
DD transplant (prior dialysis)				56 (20)
LD transplant (prior dialysis)				94 (34)
Pre-emptive transplant				128 (46)
Age (yrs), mean (SD), *missing n* = *1*	53 (10)	54 (10)	53 (10)	52 (11)
Gender (male)	339 (53)	148 (50)	33 (59)	158 (56)
Educational level, *missing n* = 4				
Primary education	213 (34)	105 (35)	23 (42)	85 (31)
Secondary education	208 (33)	100 (34)	17 (31)	91 (33)
Tertiary education	209 (33)	92 (31)	15 (27)	102 (37)
Ethnicity, *missing n* = *5*				
Dutch	586 (93)	278 (94)	51 (93)	257 (92)
Other^a^	43 (7)	18 (6)	4 (7)	21 (8)
Employment status				
Employed	409 (65)	202 (68)	29 (52)	178 (64)
Work disability status (benefits)^b^	223 (35)	86 (29)	37 (66)	100 (36)
Financial situation, *missing n* = *8*				
Shortage of money	81 (13)	30 (10)	9 (17)	42 (15)
Making ends meet	132 (21)	76 (26)	11 (21)	45 (16)
Having money left over	413 (66)	188 (64)	33 (62)	192 (69)
Primary kidney disease				
Renal vascular disease/diabetes	91 (14)	41 (14)	14 (25)	36 (13)
Glomerulonephritis	49 (8)	24 (8)	2 (4)	23 (8)
Polycystic kidney disease	141 (22)	65 (22)	4 (7)	72 (26)
Other^c^	235 (37)	118 (40)	25 (45)	92 (33)
Unknown	118 (19)	50 (17)	11 (20)	57 (20)
Comorbidity				
No comorbid conditions	231 (36)	94 (32)	13 (23)	124 (44)
1 comorbid condition	202 (32)	99 (33)	22 (39)	81 (29)
≥ 2 comorbid conditions	201 (32)	105 (35)	21 (38)	75 (27)
Health status (KDQoL),^d^ mean (SD)				
General health, *missing n* = *8*	42.5 (23.0)	38.3 (22.3)	25.5 (19.9)	50.3 (21.3)
Physical functioning, *missing n* = *6*	64.3 (34.1)	61.1 (34.2)	41.1 (34.1)	72.3 (31.3)
Mental functioning, *missing n* = *7*	62.7 (20.0)	59.5 (19.2)	53.8 (20.5)	67.8 (19.6)
Fatigue (CIS),^e^ mean (SD), *missing n* = *12*				
Subjective fatigue	31.6 (13.9)	34.7 (13.4)	38.9 (11.7)	26.9 (13.3)
Cutoff (> 35) severe fatigue	264 (42)	153 (52)	37 (69)	74 (27)

Data presented as *n* (percent) unless otherwise indicated. Percentages are column percentages and may not add up to 100% due to rounding. If data were missing, the number is presented

*KRT* kidney replacement therapy, *DD* deceased donor, *LD* living donor, *KDQoL* Kidney Disease Quality of Life-Short Form, *CIS* Checklist Individual Strength

^a^Participants born abroad, or one or both parents born abroad

^b^Categories are not mutually exclusive as in the Netherlands, work disability benefits can be either full or partial. Employed patients can also receive supplementary work disability benefits

^c^E.g., side effects of medication, accident

^d^Higher scores indicate better general health and functioning

^e^Higher scores indicate more fatigue

### Employment and work disability status

Sixty-five percent were employed and 35% were work disabled (Table 1). Of the dialysis patients, 52% had a paid job, compared to 68% of the CKD3b-G5 patients and 64% of the transplant patients. Of the non-employed, 61% were work disabled, 13% were looking for a job, 13% had retired early, and 11% never had a job (data not shown).

### Differences between employed and non-employed patients

Table 2 reports socio-demographic, clinical, and health status characteristics for employed and non-employed patients. Employed patients were younger, were more often male, more often had a higher educational level, were less often receiving work disability benefits, and less often had a shortage of money. Employed patients also had fewer comorbidities, better health status, and less fatigue (37% vs. 53%).

**Table 2:** Socio-demographic, clinical, and health status characteristics stratified by employment status

Characteristic	All patients (*n* = 634)	Employed (*n* = 409)	Non-employed (*n* = 225)	*P* values
Age (yrs), mean (SD), *missing n* = *1*	53.4 (10.3)	51.7 (10.4)	56.3 (9.5)	< 0.001
Gender (% male)	339 (53)	242 (59)	97 (43)	< 0.001
Educational level, *missing n* = *4*				< 0.001
Primary education	213 (34)	105 (26)	108 (48)	
Secondary education	208 (33)	142 (35)	66 (30)	
Tertiary education	209 (33)	160 (39)	49 (22)	
Ethnicity, *missing n* = *5*				0.79
Dutch	586 (93)	381 (93)	205 (93)	
Other^a^	43 (7)	27 (7)	16 (7)	
Work disability status (benefits)	223 (35)	86 (21)	137 (61)	< 0.001
Financial situation, *missing n* = *8*				< 0.001
Shortage of money	81 (13)	30 (7)	51 (23)	
Making ends meet	132 (21)	67 (17)	65 (30)	
Having money left over	413 (66)	309 (76)	104 (47)	
Primary kidney disease				0.04
Renal vascular disease/diabetes	91 (14)	51 (12)	40 (18)	
Glomerulonephritis	49 (8)	39 (10)	10 (4)	
Polycystic kidney disease	141 (22)	98 (24)	43 (19)	
Other^b^	235 (37)	148 (36)	87 (39)	
Unknown	118 (19)	73 (18)	45 (20)	
CKD Stage and KRT				0.07
G3b-G5	298 (47)	202 (49)	96 (43)	
Dialysis	56 (9)	29 (7)	27 (12)	
Transplantation	280 (44)	178 (44)	102 (45)	
Dialysis				0.15
Hemodialysis	43 (77)	20 (69)	23 (85)	
Peritoneal dialysis	13 (23)	9 (31)	4 (15)	
Transplantation, *missing n* = *2*				0.002
DD transplant (prior dialysis)	56 (20)	26 (15)	30 (30)	
LD transplant (prior dialysis)	94 (34)	58 (33)	36 (36)	
Pre-emptive transplant	128 (46)	94 (53)	34 (34)	
Comorbidity				< 0.001
No comorbidities	231 (36)	182 (44)	49 (22)	
1 comorbid condition	202 (32)	129 (32)	73 (32)	
≥ 2 comorbid conditions	201 (32)	98 (24)	103 (46)	
Health status (KDQoL)^c^, mean (SD)				
General health, *missing n* = *8*	42.5 (23.0)	48.2 (21.1)	32.2 (22.7)	< 0.001
Physical functioning, *missingn* = *6*	64.3 (34.1)	73.1 (29.4)	48.3 (36.1)	< 0.001
Mental functioning, *missing n* = *7*	62.7 (20.0)	65.3 (18.5)	52.0 (21.7)	< 0.001
Fatigue (CIS)^d^, mean (SD), *missing n* = *12*				
Subjective fatigue	31.6 (13.9)	29.7 (13.5)	35.1 (13.9)	< 0.001
Cutoff (> 35) severe fatigue	264 (42)	147 (37)	117 (53)	< 0.001

Data presented as *n* (percent) unless otherwise indicated. Percentages may not add up to 100% due to rounding. If data were missing, the number is presented

CKD, chronic kidney disease; KRT, kidney replacement therapy; DD, deceased donor; LD, living donor; KDQoL, Kidney Disease Quality of Life-Short Form; CIS, Checklist Individual Strength

^a ^Participants born abroad, or one or both parents born abroad

^b^ E.g., side effects of medication, accident

^c^ Higher scores indicate better general health and functioning

^d^ Higher scores indicate more fatigue

Regarding kidney replacement therapy, compared to non-employed transplant patients, employed transplant patients more often received a pre-emptive transplantation and less often a deceased transplantation with prior dialysis. Although among employed dialysis patients the rate of peritoneal dialysis was higher, it was not statistically significant.

### Associations of sustained employment with socio-demographic, clinical and health characteristics

Univariable regression analysis (Table 3) showed significant associations of sustained employment with age, gender, education, dialysis, deceased donor transplantation, renal vascular disease/diabetes, comorbid conditions, health status and fatigue.

**Table 3:** Associations of socio-demographic, clinical, and health status characteristics with employment status (*N* = 576)

	Univariable OR (95% CI)	*P* values	Multivariable^a^ OR (95% CI) *N* = 567	*P* values	Multivariable^b^ OR (95% CI) *N* = 565	*P* values
*Socio-demographic characteristics*						
Age (18–34 yrs, ref)						
35–44 yrs	0.15 (0.03 to 0.67)	0.01	0.21 (0.04 to 1.09)	0.06	0.17 (0.04 to 0.84)	0.03
45–54 yrs	0.20 (0.05 to 0.86)	0.03	0.28 (0.06 to 1.33)	0.11	0.25 (0.05 to 1.17)	0.08
55–64 yrs	0.09 (0.02 to 0.39)	0.001	0.14 (0.30 to 0.64)	0.01	0.12 (0.03 to 0.53)	0.01
Gender (male, ref)						
Female	0.52 (0.36 to 0.75)	< 0.001	0.51 (0.33 to 0.78)	0.002	0.49 (0.32 to 0.75)	0.001
Education (primary, ref)						
Secondary	2.15 (1.40 to 3.31)	0.001	1.46 (0.88 to 2.42)	0.14	1.60 (0.98 to 2.61)	0.06
Tertiary	3.63 (2.27 to 5.81)	< 0.001	2.62 (1.53 to 4.49)	< 0.001	2.75 (1.63 to 4.62)	< 0.001
Ethnicity (Dutch, ref)						
Other	1.22 (0.56 to 2.67)	0.62				
*Clinical characteristics*						
CKD stage and KRT (G3b-G5, ref)						
Dialysis	0.49 (0.26 to 0.92)	0.03	0.69 (0.33 to 1.42)	0.31	0.48 (0.24 to 0.97)	0.04
Transplantation	0.86 (0.59 to 1.26)	0.43	0.40 (0.25 to 0.64)	< 0.001	0.48 (0.30 to 0.76)	0.002
Dialysis (hemodialysis, ref)						
Peritoneal dialysis	2.70 (0.63 to 11.55)	0.18				
Transplantation (DD, ref)						
LD transplant	1.44 (0.70 to 2.98)	0.33				
Pre-emptive transplant	3.29 (1.56 to 6.92)	0.002				
Primary kidney disease (renal vascular disease/diabetes, ref)						
Glomerulonephritis	2.77 (1.14 to 6.73)	0.024				
Polycystic kidney disease	1.86 (1.01 to 3.43)	0.047				
Other	1.24 (0.72 to 2.12)	0.44				
Unknown	1.30 (0.70 to 2.40)	0.41				
Comorbidity (no, ref)						
1 comorbid condition	0.38 (0.23 to 0.62)	< 0.001			0.60 (0.35 to 1.04)	0.07
≥ 2 comorbid conditions	0.20 (0.12 to 0.32)	< 0.001			0.33 (0.19 to 0.57)	< 0.001
*Health status characteristics*						
Health status (KDQoL)^c^						
General health	1.04 (1.03 to 1.05)	< 0.001	1.03 (1.02 to 1.05)	< 0.001		
Physical health	1.03 (1.02 to 1.03)	< 0.001	1.02 (1.01 to 1.02)	< 0.001		
Mental health	1.03 (1.02 to 1.04)	< 0.001				
Fatigue (CIS)						
Subjective fatigue^d^	0.96 (0.94 to 0.97)	< 0.001			0.96 (0.95 to 0.98)	< 0.001

OR > 1 expresses higher odds of being employed

Patients who were full-time students, early retired or those who never had paid work were excluded

*OR* Odds ratio, *CI* confidence interval (95%), *Ref* Reference group, *DD* deceased donor, *LD* living donor, *KRT* kidney replacement therapy, *KDQoL* Kidney Disease Quality of Life-Short Form, *CIS* Checklist Individual Strength

^a^Multivariable model including health status

^b^Multivariable model including comorbidities

^c^Range: 0–100

^d^Range: 8–56

Due to a significant correlation between comorbidity and health status (Spearman’s Rho: − 0.40; *p* < 0.001), we built two multivariable models: one with health status and one with comorbidity. Both final models showed significant associations with employment status for advanced age (55–64 yr, OR 0.14; 95% confidence interval (CI) 0.30–0.64 and OR 0.12; CI 0.03–0.53), being female (OR 0.51; CI 0.33–0.78 and OR 0.49; CI 0.32–0.75), and higher education (tertiary education, OR 2.62; CI 1.53–4.49 and OR 2.75; CI 1.63–4.62). Compared to stage G3b-G5 patients, transplant patients had lower odds of being employed (OR 0.40; CI 0.25–0.64 and OR 0.48; CI 0.3–0.76). In the model including comorbidity, dialysis patients had lower odds of being employed compared to stage G3b-G5 patients (OR 0.48; CI 0.24–0.97) as well. In the model including health status, better general (OR 1.03; CI 1.02–1.05) and better physical health (OR 1.02; CI 1.01–1.02) indicated higher odds of being employed. Comorbidity was deleted in the last step of this final model because of the weakest and non-significant association (1 comorbid condition OR 0.77; CI 0.43–1.37; 2 comorbid conditions, OR 0.52; CI 0.29–0.94; P 0.08). However, in the model without health status, having two or more comorbid conditions and fatigue indicated lower odds of being employed (OR 0.33; CI 0.19–0.57 and OR 0.96; CI 0.95–0.98, respectively).

### Work-related characteristics, work functioning and work environment of employed patients

Table 4 shows that of the employed patients, 71% worked according to contract, whereas 21% received supplementary work disability benefits and 8% were on sick leave. The majority had a full-time job and predominantly mentally demanding tasks.

**Table 4:** Work-related characteristics, work functioning and work environment of employed patients, stratified by CKD stage

	All Patients (*n* = 409)	Stage G3b-G5 (*n* = 202)	Dialysis (*n* = 29)	Transplantation (*n* = 178)	*P* values
*Work-related characteristics*					
Work situation (%)					< 0.001
Working according to contract	291 (71)	157 (78)	7 (24)	127 (71)	
Supplementary work disability benefits	86 (21)	30 (15)	16 (55)	40 (23)	
On sick leave	32 (8)	15 (7)	6 (21)	11 (6)	
Working hours per week (%), *missing n* = *17*					0.005
< 12 h/week (minor job)	24 (6)	8 (4)	6 (23)	10 (6)	
12–29 h/week (part-time job)	134 (34)	65 (34)	7 (27)	62 (36)	
≥ 30 h/week (full-time job)	234 (60)	121 (62)	13 (50)	100 (58)	
Job demands (%), *missing n* = *11*					0.28
Physically demanding tasks	76 (19)	35 (18)	9 (31)	32 (19)	
Mentally demanding tasks	198 (50)	102 (52)	9 (31)	87 (51)	
Physically and mentally demanding tasks	124 (31)	60 (31)	11 (38)	53 (31)	
*Work functioning*					
Work ability (WAI)^a^, mean (SD), *missing n* = *28*	7.8 (4.1)	7.4 (2.2)	5.4 (2.9)	8.2 (1.9)	< 0.001
Productivity loss (QQ, hr/day), mean (SD), *missing n* = *26*	2.0 (1.9)	1.8 (1.8)	3.2 (2.4)	2.0 (1.9)	0.004
Limitations in work performance (yes, %), *missing n* = *1*	200 (49)	101 (50)	26 (90)	73 (41)	< 0.001
Type of limitations (%)^b^					
Able to work, despite some symptoms	84 (21)	46 (23)	4 (14)	34 (19)	0.45
Has to work more slowly or adapt work	62 (15)	37 (18)	8 (28)	17 (10)	0.01
Has reduced working hours	70 (17)	31 (15)	9 (31)	30 (17)	0.11
Not able to work at all	20 (5)	8 (4)	6 (21)	6 (3)	< 0.001
Expectation to work at 6 months, *missing n* = *9*					< 0.001
Yes	344 (86)	171 (86)	16 (59)	157 (91)	
Maybe	29 (7)	15 (8)	5 (19)	9 (5)	
No	27 (7)	14 (7)	6 (22)	7 (4)	
*Work environment*					
Adjustment of work (yes, %), *missing n* = *9*	105 (26)	48 (24)	14 (54)	43 (25)	0.004
Provided work accommodations (%)^c^					
Fewer weekly working hours	54 (51)	23 (48)	9 (64)	22 (51)	–
Adjustment of work schedule	25 (24)	11 (23)	4 (29)	10 (23)	–
Partially or fully working from home	20 (19)	13 (27)	1 (7)	6 (14)	–
Adjustment of work tasks	32 (31)	16 (33)	2 (14)	14 (33)	–
Change of position	18 (17)	9 (19)	1 (7)	8 (19)	–
Working at a slower pace	44 (42)	21 (44)	9 (64)	14 (33)	–
Other^d^	41 (39)	25 (52)	2 (14)	7 (16)	–
Interpersonal relations (DPQ),^e^ mean (SD)					
Social support from colleagues, *missing n* = *34*^f^	67.7 (23.6)	63.1 (24.9)	73.6 (20.4)	72.0 (21.6)	0.001
Social support from management, *missing n* = *67*^f^	67.2 (25.8)	65.0 (26.3)	66.0 (29.5)	69.5 (25.8)	0.29
Recognition, *missing n* = *19*	73.9 (23.2)	71.2 (23.4)	79.2 (26.2)	76.3 (22.1)	0.02
Reactions to the work situation (DPQ),^e^ mean (SD)					
Experience of meaning at work, *missing n* = *17*	79.2 (18.4)	79.5 (17.9)	83.1 (20.5)	78.5 (18.8)	0.52
Psychosocial work environment, *missing n* = *10*	79.1 (15.7)	78.4 (15.0)	78.5 (23.3)	80.1 (15.2)	0.29
Job satisfaction, *missing n* = *9*	81.4 (14.5)	81.4 (13.7)	81.5 (21.1)	81.4 (14.3)	0.69
Conflict work-private life, *missing n* = *12*	26.7 (24.9)	31.1 (26.5)	23.4 (19.3)	23.3 (23.0)	0.002

Data presented as *n* (percent) unless otherwise indicated. Percentages may not add up to 100% because of rounding. If data were missing, the number is presented

*WAI* Work Ability Index, *QQ* Quantity and Quality questionnaire, *DPQ* Danish Psychosocial Work Environment Questionnaire

^a^Higher scores indicate higher work ability

^b^Multiple response options possible

^c^Multiple response options possible; percentages are based on those who reported any work adjustment; due to small sample size of subgroups, differences between groups not statistically tested

^d^For example: transfer to another department, adjustments in the workplace, accessibility of the workplace

^e^Higher scores indicate higher levels of presented dimensions

^f^This item is not applicable for many self-employed participants

Regarding work functioning, patients had moderate work ability (mean WAI 7.8) and reported a mean productivity loss of 2.0 h/day. Forty-nine percent experienced CKD-related limitations in their work and 7% expected to drop out of the workforce within the following 6 months.

Twenty-six percent of the patients had made some adjustments in their work. Frequently provided accommodations were: working fewer hours (51%), working at a slower pace (42%), adjustment of work tasks (31%) or work schedule (24%), working from home (19%), and change of position (17%). Patients scored relatively high on meaning at work, psychosocial environment and job satisfaction (mean ranges 79.1–81.4).

### Differences between CKD stages in work characteristics, work functioning and work environment

Comparison between employed Stage G3b-G5, dialysis, and transplant patients showed that dialysis patients were more often partially work disabled (55%) and on sick leave (21%), and more often had minor or part-time jobs (Table 4).

Regarding work functioning, transplant patients reported the highest level of work ability (mean 8.2; ‘good’), experienced the least limitations in work performance (41%), and 91% expected to maintain work in 6 months. Dialysis patients had ‘poor’ work ability (mean 5.4), the highest productivity loss and highest proportion of patients (90%) experiencing limitations, as well as the highest proportion that expected to drop out of the workforce (22%).

Results on work environment showed the greater need for work adjustment among dialysis patients (54%). Compared to dialysis and transplant patients, stage G3b-G5 patients reported less social support from colleagues (mean 63.1) and these patients perceived more conflict between work and private life (mean 31.1).

In order to check for potential confounding of age, gender and educational level, we built adjusted models for continuous variables (see Supplementary File), which showed that all statistically significant associations listed in Table 4 remained significant.

## Discussion

This study investigated sustained employment in Stage G3b-G5, dialysis, and transplant patients. Two thirds of the study group had a paid job (60% full-time). However, 21% of these working patients received supplementary work disability benefits, and in total, one third of the study group was (partially) work disabled. Employed patients had better health although one third reported severe fatigue. Half of employed patients experienced CKD-related limitations at work and one in four patients needed some work adjustments. Non-employed patients experienced financial hardship, underlining the importance of employment for maintaining an income. Multivariable regression analysis showed associations of younger age, male gender, higher education, better health status, and early/late-stage CKD (CKD G3b-G5) with being employed. Dialysis patients reported the greatest limitations and work adjustments. Stage G3b-G5 patients experienced the lowest level of support from colleagues and felt more conflict in their work- and private life. Regarding transplantation, pre-emptive transplant patients had the highest employment rate.

Compared to the 69% employment rate in the general Dutch population [22], Sage G3b-G5 patients have a similar employment rate (68%), whereas transplantation patients (64%) and dialysis patients (52%) have lower rates. Although comparison is difficult because of variability in measurement of employment and in characteristics of the study participants across studies and countries, available literature shows high variability in employment rates of CKD patients (range 18–82%) [3, 23, 24] as is the case in patients with other chronic conditions (range 14–75%) [6, 25]. Compared to the 6% work disability rate in the general population [25], we found relatively high disability rates among patients (i.e., stage G3b-G5 29%; dialysis 66%; transplantation 36%), which is in line with previous studies [1, 4, 26–28].

In addition to employment and work disability status, our study focused on how employed CKD patients function at work. We found slightly lower work ability levels (mean WAI 7.8, SD 4.1) than those reported in previous studies among general working populations (mean 8.2, 95% CI 8.2–8.4 [27]; mean 7.95 ± 1.2 to 8.48 ± 1.4 [28]). However, of the employed patients in our study, half reported limitations at work due to CKD, a lower percentage than in a previous Dutch study among CKD patients (85%)[6].

The present study showed multiple associations between patient characteristics and sustained employment. Concerning transplantation, pre-emptive transplantation is positively associated with employment, potentially explained by avoidance of the harmful effects of dialysis on general health [29], and its subsequent risk of job loss. The multivariable models indicated social and gender inequalities; more poorly educated patients and women were at higher risk of unemployment. Moreover, poor health increased the risk of unemployment. With regard to CKD stage, transplant patients have lower odds of being employed compared to stage G3b-G4 patients. This indicates that concerning work participation, transplant patients are at a disadvantage compared to patients before the start of kidney replacement therapy. We found that 23% of transplant patients still depended on work disability benefits. This indicates decreased work ability and persistent functional limitations in transplant patients, also found in previous studies [4].

A remarkable result was that 50% of the employed Stage G3b-G5 patients experienced work performance limitations and 24% adopted work accommodations such as reducing working hours, working at a slower pace, and adjusting work tasks. Seven percent expected to drop out of the workforce, and another 8% were uncertain about maintaining their work in six months. Moreover, patients experienced the lowest level of support from colleagues. In its early stages, CKD is often an invisible condition that may be poorly understood by others [30]. At the workplace, patients often avoid or postpone disclosure of their CKD out of fear of stigmatization and prejudice, and worries about job loss [31]. Interestingly, stage G3b-G5 patients more often perceived conflict between work- and private life compared to dialysis and transplant patients; their private life suffered under the amount of energy and time they spent at work. Clearly, these patients struggle to find an acceptable work-life balance. Early employment-related counseling may support patients [12, 32].

Dialysis patients appeared particularly vulnerable concerning employment and work functioning [1]. Compared to Stage G3b-G5 and transplant patients they reported low work ability, more sickness absence and supplementary work disability benefits, more productivity loss, as well as greater need for work adjustments. Autonomy and job control (i.e., the possibility to plan and pace work tasks [8]), work accommodations [33], and dialysis scheduling around work [8] can support patients in remaining productive workers.

Our study expands the knowledge on the impact of CKD on the work life of patients, which is an innovative research topic in nephrology. The comprehensive assessment of work functioning informs clinicians on work situations and limitations in work performance. The range of identified work accommodations shows the patients’ opportunities of handling work-related limitations. Another strength of this study is the inclusion of patients in CKD stage G3b-G5, a group often neglected in studies concerning the impact of kidney failure on patients’ lives.

A study limitation is the convenience sampling in nine nephrology, dialysis, and transplantation departments; this did not allow for a central tracking system with an overview of eligible patients and characteristics in all participating centers. Consequently, reasons for non-participation were not registered and may be a source of bias. Moreover, as we lacked information about CKD patients who were not willing to participate we were unable to compare responders with non-responders. As a consequence, it is not possible to conclude that our study sample is representative of all CKD patients and might overestimate the employment rates of the overall CKD patient population. As some previous studies in the Netherlands [1] showed lower employment rates among CKD patients, and others showed similar rates [4, 6], our study may overestimate employment status and underestimate limitations in work functioning and work disability. In addition, our sample tends to be better educated which generally is associated with higher employment rates. Furthermore, the large proportion of pre-emptive and living donor transplant patients, and patients with polycystic kidney disease, as well as the relatively low proportion of diabetes as primary disease that is common for the Netherlands, may limit generalizability of findings to other countries with different patient characteristic distributions. The relatively small sample size of dialysis patients, with a small proportion of peritoneal dialysis, restricted statistical testing of some variables. In addition, we did not collect data on occupational type or occupational position. Possibly, patients from more socially disadvantaged positions may experience greater socioeconomic impact of CKD. Future research must show whether work impact varies across occupational positions.

In conclusion, although work participation is feasible for many CKD patients, they often experience substantial limitations in work functioning that require adjustment of work or partial work disability, combined with a part-time job. Patients with early/late-stage CKD struggle with their work-life balance and support at work, and in transplant patients work functioning is not completely restored. Therefore, clinicians should communicate about their patients’ work situation, potential problems and support needs, and refer them for occupational counseling where needed. Furthermore, person-centered nephrology care, integrating CKD treatment and work, and individual tailoring of workplace accommodations may facilitate work functioning. Lastly, vocational rehabilitation programs or interventions should be developed to enable sustainable employment of CKD patients.

## Supplementary Information

Below is the link to the electronic supplementary material.

Supplementary file1 (DOCX 15 kb)

## Data Availability

The dataset generated and analyzed during the current study are available from the corresponding author on reasonable request.
