# Supplementary material for: Sustained employment, work disability and work functioning in CKD patients: a cross-sectional survey study
Source: J Nephrol. 2022 Oct 31;36(3):731–43. doi: 10.1007/s40620-022-01476-w (PMC10090013; doi:10.1007/s40620-022-01476-w)
Supplement: Supplementary file 1 — Supplementary file1 (DOCX 15 kb) [file 40620_2022_1476_MOESM1_ESM.docx]

**Supplementary Table: Work functioning and work environment of employed patients, stratified by CKD stage and adjusted for age, gender and educational level**

|  | | | **Descriptive data** | | | | | | | | | | **Unadjusted** | **Adjusted^d^** | | | | |
| --- | --- | --- | --- | --- | --- | --- | --- | --- | --- | --- | --- | --- | --- | --- | --- | --- | --- | --- |
|  | | | **All Patients**  **(*n*=409)** | | | | **Stage G3b-G5**  **(*n*=202)** | | **Dialysis**  **(*n*=29)** | **Transplantation**  **(*n*=178)** | | | ***P* values** | **F (2,5)** | | ***P* values** | | |
| **Work functioning** | | |  | | | |  | |  |  | | |  |  | |  | | |
| Work ability (WAI)^a^, mean (SD), *missing n=28* | | | 7.8 (4.1) | | | | 7.4 (2.2) | | 5.4 (2.9) | 8.2 (1.9) | | | <.001 | 5.458 | | .005 | | |
| Productivity loss (QQ, hr/day), mean (SD), *missing n=26* | | | 2.0 (1.9) | | | | 1.8 (1.8) | | 3.2 (2.4) | 2.0 (1.9) | | | .004 | 7.739 | | .001 | | |
| **Work environment** | | |  | | | |  | |  |  | | |  |  | |  | | |
| Interpersonal relations (DPQ),^b^ mean (SD) | | |  | | |  | |  | | |  |  | | |  | |  |  |
|  | Social support from colleagues, *missing n=34*^c^ | | 67.7 (23.6) | | | | 63.1 (24.9) | | 73.6 (20.4) | 72.0 (21.6) | | | .001 | 7.004 | | .001 | | |
|  | Social support from management, *missing n=67*^c^ | | 67.2 (25.8) | | | | 65.0 (26.3) | | 66.0 (29.5) | 69.5 (25.8) | | | .29 | 1.008 | | .37 | | |
|  | Recognition, *missing n=19* | | 73.9 (23.2) | | | | 71.2 (23.4) | | 79.2 (26.2) | 76.3 (22.1) | | | .02 | 3.059 | | .048 | | |
| Reactions to the work situation (DPQ),^b^ mean (SD) | |  | |  |  | | | |  |  | | |  |  | |  | | |
|  | Experience of meaning at work, *missing n=17* | | 79.2 (18.4) | | | | 79.5 (17.9) | | 83.1 (20.5) | 78.5 (18.8) | | | .52 | 0.753 | | .47 | | |
|  | Psychosocial work environment, *missing n=10* | | 79.1 (15.7) | | | | 78.4 (15.0) | | 78.5 (23.3) | 80.1 (15.2) | | | .29 | 0.498 | | .61 | | |
|  | Job satisfaction, *missing n=9* | | 81.4 (14.5) | | | | 81.4 (13.7) | | 81.5 (21.1) | 81.4 (14.3) | | | .69 | 0.039 | | .64 | | |
|  | Conflict work-private life, *missing n=12* | | 26.7 (24.9) | | | | 31.1 (26.5) | | 23.4 (19.3) | 23.3 (23.0) | | | .002 | 7.185 | | .001 | | |

Data presented as *n* (percent) unless otherwise indicated. Percentages may not add up to 100% because of rounding. If data were missing, the number is presented.

WAI, Work Ability Index; QQ, Quantity and Quality questionnaire; DPQ, Danish Psychosocial Work Environment Questionnaire.

^a^Higher scores indicate higher work ability.

^b^Higher scores indicate higher levels of presented dimensions.

^c^This item is not applicable for many self-employed participants.

^d^Analysis of covariance Models adjusted for age, gender and educational level.
